# Do long-term care services match population needs? A spatial analysis of nursing homes in Chile

**DOI:** 10.1371/journal.pone.0199522

**Published:** 2018-06-26

**Authors:** Pablo Villalobos Dintrans

**Affiliations:** Harvard T. H. Chan School of Public Health, Boston, Massachusetts, United States of America; University of Georgia, UNITED STATES

## Abstract

Chile is experiencing a period of rapid aging, which increases the need of long-term care services in the country. Nursing homes have been the traditional alternative to deal with the increase of elderly population in the country, with services supplied by a mix of for-profit and nonprofit providers. Additionally, population exhibits a high degree of geographical concentration. The study aims to identify the determinants of the geographical location of nursing homes in Chile at municipality level. The analysis takes into account the different location criteria for different types of nursing homes as well as potential spatial effects. The paper uses spatial analysis tools to identify clusters of nursing homes and population characteristics and to estimate the determinants of nursing homes availability and coverage in the country. The analysis–based on spatial global and local tests, and spatial autoregressive models- show the existence of clusters of nursing homes as well as clusters of municipalities according to elderly population, income, poverty, population density, and public health insurance coverage. Residuals from ordinary least squares regressions were spatially autocorrelated, showing the need of using spatial models. Estimations show that availability and coverage of nursing homes are positively related with municipality income, and that for-profit and nonprofit facilities respond differently to different factors. A negative coefficient was found for poverty in nonprofit nursing homes, raising doubts about the effectiveness of giving public subsidies to incentive the installation of facilities in areas with high needs and low income.

## Introduction

Chilean population is getting older and increasingly dependent. By 2038, the share of population over 65 years will almost double, going from 11% in 2017 to 20% [1]. Physical and mental health problems rise significantly from 65 years old, being particularly prevalent for people older than 85 years, increasing the need of long-term care (LTC) services in the population [2,3]. Estimations for Chile show, for example, that the burden of mental diseases and dependency is expected to triple in the next 35 years [4].

LTC is defined as a range of services required by persons with a reduced degree of functional capacity (physical or mental), and who are dependent for an extended period of time on help with basic activities of daily living [5]. In Chile, the main LTC policy has been to subsidize the supply of LTC services, via funding nursing homes (NH). Currently, these services are provided by a mix of private for-profit, private not-for-profit, and public facilities, many of them receiving public subsidies.

Population in the country tends to agglomerate. People cluster based on socioeconomic and demographic characteristics, such as income and age. Several explanations are possible to explain this phenomenon, ranging from sociological justifications (e.g. homophily, discrimination) to economic reasons (e.g. housing market, availability of amenities, work places). Residential patterns exhibit uniformity, because people try to maintain homogeneous communities and pay more to live in homogeneous neighborhoods [6–8]. For Chile, several studies confirm these patterns of residential segregation and geographical concentration of income [9–12].

Considering the features of the Chilean nursing homes market–mix of for-profit and not-for-profit provides, and public funding–it is expected that different providers choose the location of the nursing homes based on different attributes. On the one hand, public funds are allocated into not-for-profit and public facilities, subject to meeting certain eligibility criteria: age (over 60 years old), socioeconomic condition (social vulnerability), and coverage by the National Health Fund (*Fondo Nacional de Salud*, FONASA), the public health insurance [13]. On the other hand, private institutions decide to enter the market based on profit-maximization criterion. The standard spatial competition models [14,15] assume that firms decide on the number of facilities, their location and the price. The supply is influenced by the size of the market, willingness to pay, cost of opening a facility, and transportation costs. In markets where location is a decision variable, two opposite forces operate: on the one hand, all the firms want to be where the demand is, which implies that we would observe firm concentration in specific market, according to the principle of minimum differentiation [16, 17]. On the other hand, location helps firms to differentiate from each other and avoid price competition [18, 19]. Under this scenario, firms should be apart from each other, following the principle of maximum differentiation [16, 19].

Following the eligibility requirement to get public funds and the literature on spatial competition, it is possible to assume that choices regarding for-profit and nonprofit NH come from different decision-making processes:

$${For-profit:\;max}_{N,T,P,L}\;U^{fp}=f\;(age,income,other\;features)$$ (1)

$$Nonprofit:\;{max}_{N,T,P,L}\;U^{np}=g\;(age,income,other\;features)$$ (2)

where the control variables are the number of NH (N), the size of each NH (T), and the price charged (P). All these choices are mediated by the decision on location (L), i.e. where these nursing homes will be operating. The functional forms–*f* and *g*- define the way in which different attributes of a given region influence utility for both types of firms.

For-profit NH are expected to act as profit-maximizers. In this case, Eq 1 can take the specific form:

π=pq−(cq+F) (3)

where π represents the profits, *q* is the quantity of services sold, *p* is the price charged, *c* is the marginal cost of producing the services and *F* are fixed costs of operation. In this case, age, income, and other features of the chosen location influence both revenues (pq) (people demanding services depend on the need for these services and ability to pay) and costs (pc+F) (housing market, labor costs, distance). On the other hand, nonprofit maximizes a social utility function that includes providing services to people in need (related to age), but also equity components (socioeconomic criterion) and practical requirements (enrollment in FONASA).

Finally, since John Snow’s seminal work [20], space and place have increasingly used to analyze and understand health decisions and outcomes. From mapping mortality and morbidity to analyze epidemics, spatial analysis has proved to be an important tool in health research and public health [21–23]. In particular, spatial regressions have been used for health planning; for example, to explain factor behind clusters of pathologies, to understand healthcare utilization, or to determine the availability of resources for and access to healthcare [24, 25].

Considering all these elements, the aim of the study is to identify the determinants of the geographical location of nursing homes in Chile, using spatial regressions to account for the effect of space and place in the determinants of facilities in the country. The analysis takes into uses the different location criteria for different types of nursing homes–for-profit and nonprofit criteria–as well as potential spatial effects arising from the fact that factors triggering the decisions about LTC services in the country are potentially spatially auto correlated.

## Data and methods

### Data sources and description

Three different sources were used in the study. First, a list of nursing homes (NH) in the country, containing information on address, ownership, number and type of people attended, price, and amenities, for the 724 facilities in the country. Data was collected by the National Elderly Service (*Servicio Nacional del Adulto Mayor*, SENAMA) (http://catastroeleam.senama.cl.). The second dataset contains demographic and socioeconomic information for the 346 municipalities in Chile, published by the Ministry of Social Development (http://observatorio.ministeriodesarrollosocial.gob.cl/indicadores/reportes_com1_2.php). Finally, maps and borders were obtained from the Ministry of National Assets (http://www.ide.cl/descarga/capas.html).

Table 1 shows the distribution of nursing homes by ownership and price range. First, it is interesting to note that 66% of the nursing homes are private for-profit, and almost half of them are in the high price category (over CLP$250,000; around US$490). The situation is different when looking at the number of people in NH: 56% of the NH population receives services from a nonprofit NH and only 36% of the people is in a NH that charges more than CLP$250,000. Second, as expected most of the high price nursing homes are for-profit, while 90% of the NH in the lowest price category are nonprofit; this pattern is similar when looking at people attended instead of number of NH.

**Table 1:** Distribution of nursing homes and number of people in NH by price and ownership (Chilean pesos, CLP$ 2010).

	For-profit	Nonprofit	Total
***# Nursing homes***			
0–50,000 (CLP$)	10	71	81
50,001–150,000 (CLP$)	68	112	180
150,001–250,000 (CLP$)	124	6	130
250,001–350,000 (CLP$)	98	10	108
Over 350,001 (CLP$)	177	48	225
**Total**	477	247	724
***Capacity (# people)***			
0–50,000 (CLP$)	178	3,748	3,926
50,001–150,000 (CLP$)	1,301	4,784	6,085
150,001–250,000 (CLP$)	2,359	170	2,529
250,001–350,000 (CLP$)	1,521	368	1,889
Over 350,001 (CLP$)	3,233	1,946	5,179
**Total**	8,592	11,016	19,608

Note: Exchange rate 2010 = 510.38 (CLP$/US$). Source: Banco Central de Chile.

Nonprofit NH includes 18 public facilities with capacity for 491 persons.

Table 2 presents statistics at country and municipality level for a group of variables of interest. Variables were selected according to the conceptual framework presented in the previous section. The main variables of interest are the coverage of LTC services–defined as the percentage of people over 65 years in NH–and the availability of LTC services–defined as the number of NH per 10,000 people over 65 years–in each municipality. Both variables capture different dimensions of the decision of opening a NH: coverage is directly related to how many people have access to LTC services in certain area, while availability considers diversity of alternatives in terms of different providers, prices and distances. For example, two areas with the same population, elderly population and coverage can be very different if in one case all the services are provided by a single NH and, in the other case, by several NH. The relevance of availability is evident in the first case if, for example, the NH is located in the center versus one extreme of the city, or if the only provider charges low versus high prices.

**Table 2:** Descriptive statistics.

	Chile	Municipality (N = 345) ^a^				
		Average	Max	Min	St. Dev.	Moran's I ^g^
(A) Population	18,005,048	52,038	610,118	121	80,917	0.32***
(B) Population 65+	1,855,434	5,363	53,668	9	7,917	0.40***
(C) % population 65+ (B)/(A)	0.103	0.113	0.20	0.02	0.03	0.41***
(D) Nursing homes	724	2.12	53.00	0 ^b^	5.72	0.37***
(E) People in nursing homes	19,608	57.33	1,300	0 ^c^	135.39	0.35***
(F) People per NH (E)/(D)	27.08	28.80 ^b^	300 b	9 ^d^	27.06 ^d^	0.07***
**(G) Coverage (E)/(B)**	0.011	0.007	0.065	0 ^e^	0.010	0.12***
(H) Availability (D)/{(B)*10,000}	3.90	2.59	32.79	0 ^f^	4.15	0.19***
(I) Poverty (% population)	14.40	15.88	48.80	0.10	7.71	0.55***
(J) Average wage (CLP$)	563,414	465,170	1,425,074	266,506	143,916	0.66***
(K) Density (people 65+ per km^2^)	0.07	115.83	2,439.45	0.001	389.18	0.76***
(L) FONASA (% population)	0.73	87.59	100.00	14.30	11.07	0.39***

Notes

^a^ Excludes Antarctica.

^b^ 164 municipalities have zero nursing home.

^c^ Minimum number of people in a nursing home is 9 in municipalities with at least one nursing home.

^d^ Calculated using municipalities with at least one nursing home.

^e^ Minimum coverage is 0.001 in municipalities with at least one nursing home.

^f^ Minimum availability is 0.278 in municipalities with at least one nursing home.

^g^ Pseudo p values using 999 permutations

*** less than 0.01.

The second set of variables comprises those identified as important in deciding where to provide LTC services. As stated before, these factors can differ between nonprofit and for-profit providers; for the former, the decision is expected to be related to government’s criteria (age, income, and health insurance), while in the case of for-profit providers the main drivers are market size (share of elderly population), willingness to pay (income, poverty), and distance to consumers (density).

First, it is important to notice the high variation of values for each variable at municipality level. For example, population in the most populated municipality is 5,000 times higher than in the least populated, the share of elderly population varies between 2% and 20%, poverty ranges from almost 0% to 50%, FONASA (public health insurance) coverage ranges from 14% in Vitacura (northeastern municipality in the Metropolitan Region) to 100% in Tirúa and Portezuelo (both in the Bio-Bio region). Second, heterogeneity is high, but values are not randomly distributed in the Chilean territory. As shown in the last column of Table 2 by the global Moran’s I, a test for special randomness that indicates both the existence and degree of spatial autocorrelation [26]. Moran’s I ranges from -1 to 1, with -1 indicating perfect dispersion and 1 as an indicator of perfect clustering. As presented in Table 2, all variables show a positive and significant value for the indicator, meaning that the hypothesis that variables are randomly distributed among municipalities is rejected in every case.

**Fig 1:**
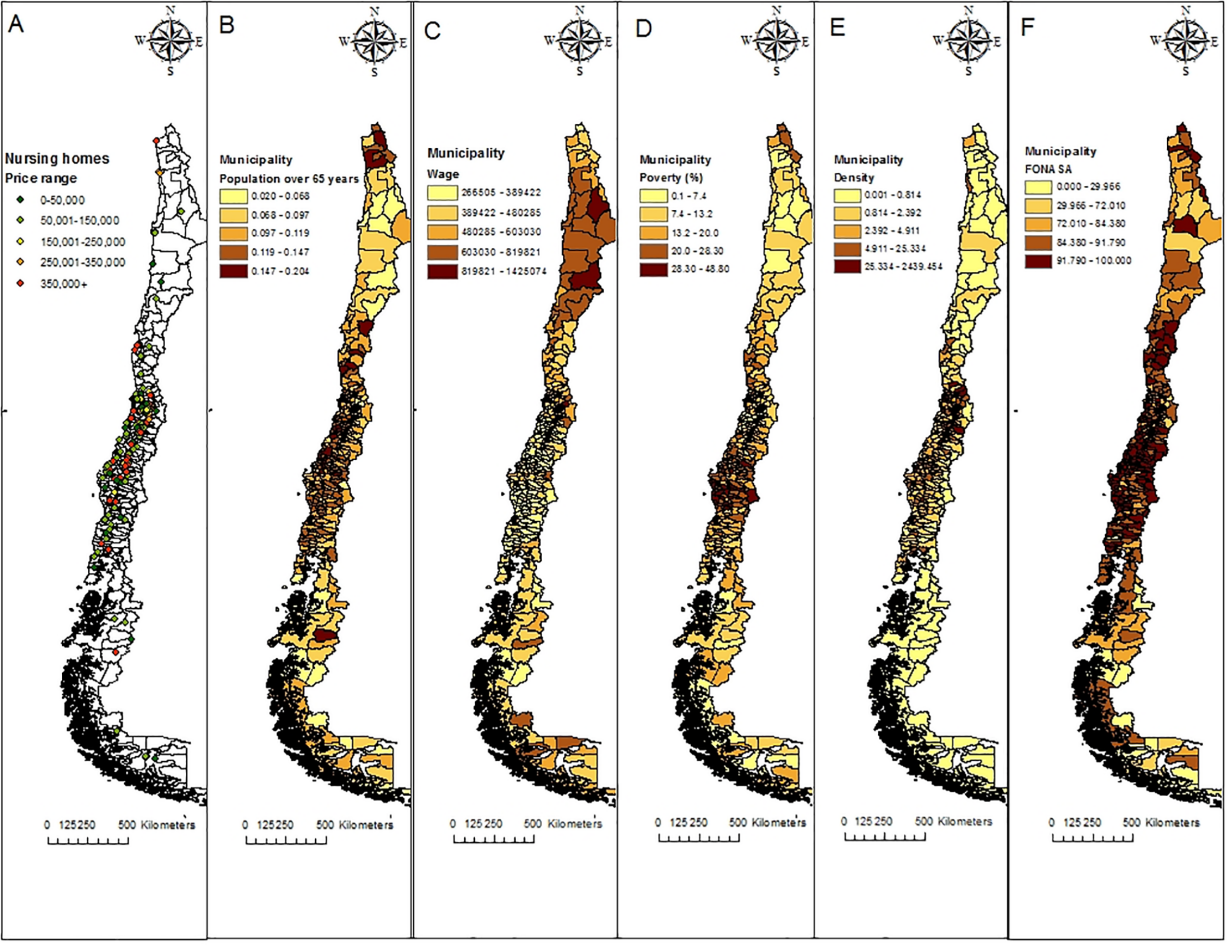
Map of nursing homes and geographical distribution of independent variables. A: Nursing homes by price range. B: Population over 65 years; C: Average wage; D: Poverty (% population); E: Elderly density (population over 65/ area); F: FONASA (% population).

Fig 1 shows these patterns (heterogeneity and spatial relationship), by exhibiting the distribution of NH along the country and quintile maps for the variables of interest. Map A shows how nursing homes are concentrated in the central zone, with almost half of them in the Metropolitan Region, area that also concentrates the most expensive facilities (62% of the NH in the highest price range are in this region). Maps B to F exhibit the huge differences at municipal level for all variables, but also how particular characteristics tend to cluster in given zones of the country. For example, extreme areas (northern and southern) have younger population, while poverty is particularly prevalent in Bio-Bio and Araucanía regions.

### Spatial tests and spatial regressions

As described by [26], spatial autocorrelation and measures can be broadly classified into global and local measures. The use of local measures is interesting to identify spatial patterns in specific locations: while global tests answer the question about the presence of spatial correlation, local tests indicate where is it. As in the case of global measures, several tests can be used to determine autocorrelation at local level [26, 27]. For this study, the G_i_* [28, 29] was used, since the goal is to identify areas were availability or coverage of nursing homes is low or high (hot and cold spots detection) respect to the global average, instead of areas were these attributes are similar or different between municipalities; this justifies the choice over other commonly used measures, such as local Moran’s I or Geary’s C [26, 30].

A common feature in the use of local measures of autocorrelation is multiple and dependent testing: because the same hypothesis is tested several times (and using similar data), statistically significant results will be found just by chance. For this reason, statistics are usually corrected to take into account this issue. The false discovery rate (FDR) correction has proven to be a better strategy for identifying meaningful clusters, compared to more conservative methods [31, 32]. G_i_* parameters were estimated in ArcMap, and FDR correction was done in Microsoft Excel.

**Fig 2:**
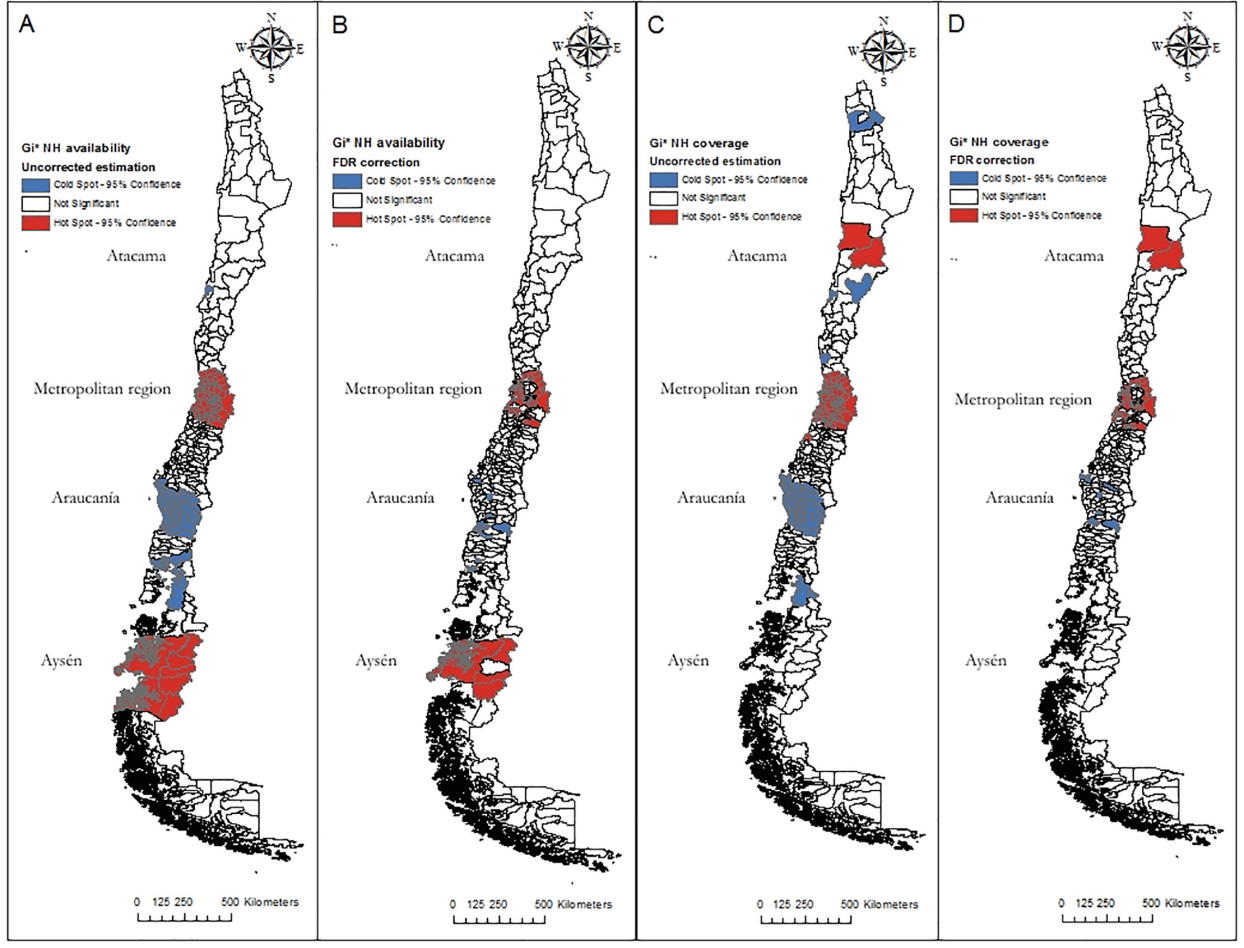
G_i_* tests for detecting clusters (NH availability and coverage). Blue: low value clusters (95% significance); Red: low value clusters (95% significance); White: not significant areas. A: NH availability, original results. B: NH availability, FDR correction; C: NH coverage, original results; D: NH availability, FDR correction.

Fig 2 presents these maps. As expected, the number of clusters decreases after applying the FDR correction, but some still remain. When looking at the number of NH (availability) high values are concentrated in the Metropolitan region and the biggest cities in Aysén (southern region); low values cluster mostly in the Araucanía region. In terms of coverage, the situation is similar, with high value clusters in the Metropolitan area and the Atacama region, and low values concentrated in the Araucanía.

In order to identify the determinants of coverage and availability of nursing homes in the country, several regressions are estimated. The unit of analysis is the municipality. Unfortunately, data on the independent variables is available only at this level of aggregation; however, decisions on location of NH and spatial correlation of independent variables are more related to smaller geographical units.

As discussed previously, the decision of open a NH (number of nursing homes) and the decision about its size (number of places offered) in a particular area depends on demographic and socioeconomic conditions, as well as other factors. This analysis is carried out by estimating several regressions, to capture the effects of several socio-demographic features on the decisions about nursing homes. In particular, the following equations are estimated using ordinary least squares (OLS):

$${Coverage}_{i}=\beta_{0}+\beta_{1}{pop65}_{i}+\beta_{2}{log(wage)}_{i}+\beta_{3}{poverty}_{i}+\beta_{4}{density}_{i}+\beta_{5}{FONASA}_{i}+\varepsilon_{i}$$ (4)

$${Availability}_{i}=\beta_{0}+\beta_{1}{pop65}_{i}+\beta_{2}{log(wage)}_{i}+\beta_{3}{poverty}_{i}+\beta_{4}{density}_{i}+\beta_{5}{FONASA}_{i}+\varepsilon_{i}$$ (5)

where *pop65* is the share of population over 65 years, *log(wage)* is the logarithm of the average wage, *poverty* is the percentage of population living in poverty, *density* is the population over 65 years divided by the area, *FONASA* is the percentage of population covered by the public insurance and ε is an error, in municipality *i*. Both equations are estimated using the total number of NH in the municipality, as well as looking at for-profit and nonprofit NH.

Given the presence of spatial autocorrelation in the data, an important concern is that coefficients from OLS regressions could be biased, because they do not take into account this feature [33]. In order to test this potential problem, OLS residuals can be examined using global Moran’s I test. If the hypothesis that OLS residuals are distributed randomly in the space is rejected, then the estimations need to be adjusted to consider the spatial effects. In this case, spatial autoregressive models were used to include spatial autocorrelation in the linear model. Two main models are commonly used: spatial lag and spatial error. The first model (also known as contagion model) incorporates space as a right hand-side variable, estimating a coefficient for the spatial effect; the error model does not incorporate spatial as a covariate, but includes it in the structure of the residuals [34]. Intuitively, both models could be appropriate for this study: on the one hand, a lag model seems suitable if we think that the decision on coverage or availability in one municipality is influenced by the number and size of NH in the neighbor municipalities, as can occur in the for-profit market for close neighbors; on the other hand, error models are adequate if unobserved spatial effects are driving the spatial autocorrelation in the residuals (e.g. factors not included in the model that are shared by neighbor municipalities). Statistical tests will be used to identify the best alternative to estimate the spatial autoregressive models. Regressions were estimated in GeoDa, using a queen contiguity matrix. Four municipalities were excluded of the analysis because they had zero neighbors (island observations): Isla de Pascua (Easter Island), Juan Fernández, Puqueldón and Guaitecas.

## Results

Eqs 4 and 5 were estimated using the total capacity (coverage) and total number of NH (availability) at municipality level. Additional estimations were run considering ownership, i.e. using coverage and capacity of for-profit and nonprofit nursing homes. Results are shown in Tables 3 and 4.

**Table 3:** Determinants of coverage: OLS and spatial error estimations (N = 341).

	OLS			Spatial Error (ML)		
	All	For-profit	Nonprofit	All	For-profit	Nonprofit
Population over 65 (%)	0.057***	0.029***	0.028	0.057**	0.024***	0.029
	(0.021)	(0.009)	(0.019)	(0.022)	(0.09)	(0.019)
Wage (log)	0.026***	0.013***	0.127**	0.032***	0.014***	0.015***
	(0.006)	(0.003)	(0.005)	(0.006)	(0.002)	(0.005)
Poverty (%)	-0.0001**	3.6e-06	-0.0001*	-7.5e-05	-1.2e-06	-9.7e-05
	(7.7e-07)	(0.002)	(6.9e-05)	(8.2e-05)	(3.5e-05)	(7.2e-05)
Density	2.3e-06	2.7e-06***	-3.8e-07	2.1e-06	2.2e-06***	-3.7e-07
	(1.55e-06)	(6.5e-07)	(1.4e-05)	(1.7e-06)	(8.1e-07)	(1.5e-06)
FONASA	4.54e-05	-1.4e-05	5.9e-05**	6.5e-05*	-8.8e-06	6.9e-05**
	(3.41e-05)	(1.4e-05)	(3.1e-05)	(3.5e-05)	(1.4e-05)	(3.1e-05)
Constant	-0.148***	-0.075***	-0.073**	-0.184***	-0.082***	-0.091***
	(0.036)	(0.015)	(0.032)	(0.039)	(0.016)	(0.034)
Moran’s I	2.887***	7.181***	1.672*	-0.001	0.001	0.001
R^2^	0.117	0.222	0.041	0.143	0.328	0.124
AIC	-2169.82	-2762.67	-2239.82	-2176.62	-2799.41	-2241.95

Significance

***1%

**5%

*10%. Standard errors in parenthesis.

**Table 4:** Determinants of availability: OLS and spatial error estimations (N = 341).

	OLS			Spatial Error (ML)		
	All	For-profit	Nonprofit	All	For-profit	Nonprofit
Population over 65 (%)	32.32***	16.868***	15.236**	34.241***	14.119***	19.056***
	(8.545)	(4.642)	(7.426)	(8.900)	(4.719)	(7.775)
Wage (log)	9.09***	6.492***	2.641	13.783***	7.475***	6.178***
	(2.40)	(1.299)	(2.078)	(2.711)	(1.459)	(2.348)
Poverty (%)	-0.063**	0.005	-0.0678**	-0.042	0.003	-0.048*
	(0.031)	(0.002)	(0.026)	(0.034)	(0.017)	(0.0292)
Density	0.0006	0.001***	-0.0008	0.0005	0.0012***	-0.0008
	(0.0006)	(0.0003)	(0.0005)	(0.0008)	(0.0004)	(0.0006)
FONASA	0.013	-0.011	0.023**	0.0378***	-0.008	0.043***
	(0.135)	(0.007)	(0.0118)	(0.013)	(0.007)	(0.012)
Constant	-52.55***	-37.038***	-15.775	-81.827***	-42.528***	-38.237***
	(14.251)	(7.742)	(12.384)	(16.056)	(8.630)	(12.919)
Moran’s I	5.691***	8.366***	4.705***	-0.009	0.002	-0.011
R^2^	0.116	0.229	0.047	0.212	0.363	0.126
AIC	1909.17	1493.01	1813.38	1882.18	1444.37	1793.70

Significance

***1%

**5%

*10%. Standard errors in parenthesis.

Table 3 shows the results for the determinants of coverage. A first set of regressions was estimated using OLS; in all cases, global Moran’s I test show the presence of spatial autocorrelation in the residuals. To deal with this problem, the same regressions were estimated using a spatial error model. As discussed before, the selection of the appropriate spatial autoregressive model was not evident; the decision was based on the statistics from OLS regressions, using Anselin’s spatial regression decision process [35]. Results of this analysis show weak evidence in favor of the error correction over the lag model: in each case the LM-lag and LM-error (not robust) are significant, as well as the robust estimators. As suggested by [35] in these cases the model should be selected using orders of magnitude, which favors the error model. These results raise doubts about potential misspecification problems, and the analysis of the specification tests (Wald > Likelihood Ratio > Lagrange Multiplier) confirms this problem, except for the estimations on nonprofit facilities.

In order to deal with this issue, the same regressions were estimated using a different weight matrix. The matrix based in distance thresholds (380 km) increases the number of neighbors for each observation, and confirms the use of an error model. In this case, specification tests do follow the expected order. The results obtained using this matrix (not shown) are very similar in terms of significance and magnitude of the coefficient. The model’s goodness of fit (R^2^) is higher for the estimations using a queen contiguity matrix, but AIC is better in estimations using only for-profit or nonprofit NH.

After running the autoregressive model, residuals are not spatially autocorrelated anymore. First, it is interesting to notice that individual coefficients are similar using both methods (OLS and spatial error), but the explained variability of the dependent variable increases largely when using the spatial autoregressive model. Second, the share of elderly population affects positively the coverage, except for nonprofit facilities; income is also related with an increase in coverage, and poverty has no effect on it. Third, density is related positively to municipal coverage of for-profit NH, but not of nonprofit facilities; on the other hand, the percentage of people covered by the public health insurance correlates with nonprofit coverage, but is not linked to capacity in for-profit NH. Except by density, all the coefficients are interpreted as the average change in the percentage of people over 65 years receiving LTC services in NH for a 1% increase in the independent variable. Finally, when looking at the different estimations (all, for-profit, nonprofit), the model does a better job explaining the variation in coverage of for-profit facilities than the total NH coverage and the nonprofit.

Table 4 shows the results for the determinants of availability. As in the case of coverage, spatial autocorrelation was present in the residuals of the OLS regressions, and the error model appeared as a better to deal with this issue. In this case, the assessment of test statistics also suggests potential misspecification problems, but the specification tests follow the expected order (Wald > Likelihood Ratio > Lagrange Multiplier).

In terms of significance, coefficients are similar to those exhibited in Table 3, except that in this case elderly population and poverty influence the availability of nonprofit facilities at municipality level. As before, R^2^ improves in all regressions when using the spatial error model, and explain better the availability of for-profit LTC facilities.

Results also hold when estimating the regression using the distance weight matrix, although R^2^ and AIC do not improve with this specification.

**Fig 3:**
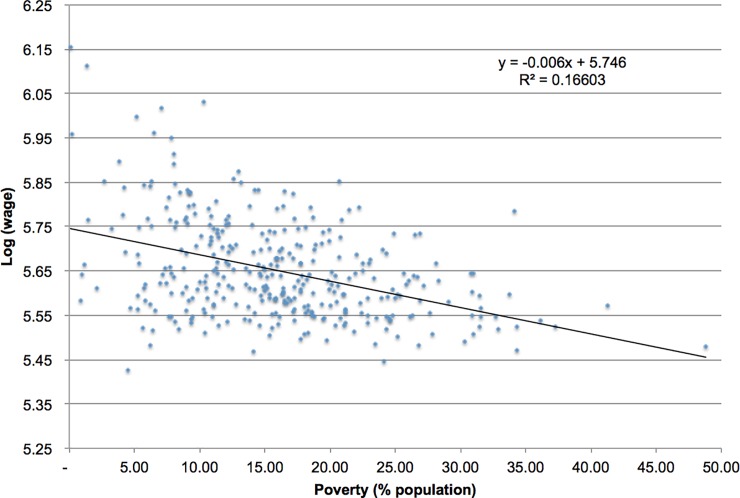
Poverty rates and wages by municipality.

In general, all coefficients have the expected sign. First, the share of elderly population is related with more and bigger NH (except for nonprofit facilities). This means that, despite ownership (and copayment), NH varies according to the size of the market. Another characteristic of the demand for LTC services is related with ability/willingness to pay. The positive correlation between wages and presence of NH was expected in for-profit facilities, but not in nonprofit ones. This effect can be explained by the existence of copayments, even for nonprofit facilities (see Table 1). In a similar way, the negative effect of poverty is surprising. The rationale for including poverty as explanatory variable is that gives information on inequality at municipal level. For example, areas with relative high wages should be attractive for private for-profit NH, but high poverty rates can be an indication of a limited demand. As shown by Fig 3, despite the fact that poverty rates are constructed using income, correlation with wages is not high. The result is unexpected not only because poverty seems to have no effect on the number and capacity of for-profit NH in a given municipality, but also because the negative effect in the case of nonprofit facilities. Estimations using price ranges instead of ownership were also estimated (not shown). In this case, a negative and significant effect was found for high-price NH, in line with the hypothesis that these facilities incorporates economic criteria in their decisions about location, but the negative effect of poverty is also present in low-price NH, showing that, in general, nursing homes are more scarce in high-poverty areas.

Density appears as important in understanding decisions only in the for-profit segment. As expected coefficients are positive, showing that these facilities are more prevalent in denser municipalities. The result can be explained from a cost perspective: densely population markets allows increasing market coverage without incurring in extra fixed costs (e.g. open a new NH), in line with an efficiency criterion. Finally, FONASA is only relevant to explain patterns in nonprofit NH, capturing some of the requirement for getting public subsidies.

Fig 3 also reveals a potential problem of heteroscedasticty, i.e. a non-constant variance of the errors. The presence of heteroscedasticity is confirmed by the Breusch-Pagan test in all estimations and, although it does not alter the coefficients, it does change the statistical inference on them. In the case of the OLS model, heteroscedasticity is somehow expected, considering that residuals are known to be spatially correlated. However, the hypothesis of constant variance in the errors is still rejected in the spatial error model, showing that, even though in this case residuals are not spatially correlated anymore, spatial effects are still present. Estimations can be improved in the OLS case by calculating coefficients’ statistics using robust standard errors; as stated before, these estimations do not change the sign and magnitude of the coefficients, but since they use a different set of errors for the inference, can alter their significance level. When using heteroscedasticity-robust standard errors in the OLS estimations most result hold but standard errors change for some variables, altering the conclusions regarding their significance levels (for example, poverty in the coverage regression using the full sample is now significant at 10% instead of 5%). In terms of the general results described before, the main change after taking into account the non-constant variance in the error matrix is that now the share of FONASA population in each municipality becomes significant in the full sample estimations (both in terms of coverage and availability). In the case of the spatial regression, the presence of heteroscedasticity indicates that the estimated model performs differently in different geographical areas. This suggests that other spatial analysis tools–particularly geographically weighted regressions (GWR)- could be useful to understand how the model performs in different settings.

## Discussion

The aim of the study is to identify the determinants of the geographical location of nursing homes in Chile. Results can be analyzed from multiple perspectives.

First, estimations for Chile show that nursing homes and beds (coverage and availability) tend to concentrates in areas of high demand, being more prevalent in municipalities with older population and larger income. Studies of nursing homes in other contexts also show that long-term care services, and particularly nursing homes, tend to agglomerate in urban and central areas, confirming that decisions about location are closely related to demand, including both economic and demographic factors [36–42].

Second, when looking at differences between for-profit and nonprofit facilities, literature finds that ownership status is associated with differences in access and results, showing that both types of facilities face different constraints and incentives, which explains differences in the way they make decision regarding geographical location, as well as other aspects [43–48]. Results from Chile confirm the hypothesis that for-profit and nonprofit nursing homes differ in the way the decision on location and capacity are made. As discussed before, estimations show that population and income attract NH in general, while FONASA coverage is important for nonprofit facilities, and density is important for for-profit NH. The model estimated works better in explaining the determinants of availability and the decisions of private for-profit firms. This analysis is relevant to understand the underlying decision-making process of private actors in the LTC services market. Both the identification of areas where gaps exist between needs and services, and the model on how firms make decisions can be useful in designing better strategies to increase access to LTC services, particularly in defining criteria for allocating public funds to new NH, defining priorities and designing policies [42, 43].

Third, studies using spatial analysis to look at more general but related issues, such as access to healthcare, find similar results in terms of the relevance of economic and demographic factors in explaining results. Although these studies answer different research questions, they are useful to understand the relevance space and place when thinking on health policies [49–53]. In the case of Chile, results highlight the need for considering the presence of spatial autocorrelation when performing an analysis at the municipality level: people of similar characteristics tend to concentrate in determined geographical areas along the country.

Despite these interesting results, it is necessary to acknowledge the study’s limitations and adjust the results’ interpretations accordingly. First, results are heavily dependents on the unit of analysis. In this case, the decision to use municipalities was influenced by the information available. Even though municipality can be the appropriate unit in many cases, it is necessary to consider that results can change when changing the scale of analysis. The study will benefit by using smaller units of analysis to capture intra-municipal variations and patterns. Second, the definition of neighborhoods is also crucial, and posed a challenge for the study. Most of the analysis was carried out using a queen contiguity matrix to identify neighbors. Again, although this strategy can be suitable in many cases, it seems less accurate for others; the immense municipal heterogeneity in terms of area and population makes it also difficult defining the “right” distance threshold. These issues raise awareness about the inherent difficulties of doing special analysis in a country like Chile. Third, one important assumption of the analysis is the relationship between population age and LTC needs. The analysis uses age as a proxy for dependency, in particular, assumes that the proportion of elderly with LTC needs is similar among different regions. This assumption can be violated if people with LTC needs tend to concentrate in specific regions (e.g. people move where supply or quality of LTC services is better). Although dependency is closely related to prevalence and level of dependency in Chile and other countries [54, 55] it is also true that a vast heterogeneity in term of long-term care needs exist among elderly [56]. This limitation could introduce bias in the estimations, but is less likely to affect those on nonprofit facilities, since public subsidies are allocated to institutions (instead of individuals), using age (not health condition or dependency) as criterion. Fourth, the analysis also makes an assumption about who is making decisions from the demand side: model relates area of residence and NH location, supposing that people (either the beneficiaries or their families) choose a facility in the same area they live. Finally, the paper gives information about quantity of NH, but ignores quality aspects, that can be relevant in providing guidance for policy-making. Considering these limitations, further research is needed. Given the heterogeneity of the data, the analysis could be extended using spatial regimes (central zone versus extreme zones) of geographically weighted regressions, to account for spatial variations in the model.

The study gives a panorama of the current situation, highlighting the need of considering both, space (location of NH) and place (socioeconomic and demographic features of the areas under analysis), when designing policies to deal with the provision of long-term care services in Chile.

## Supporting information

S1 Dataset Variables and values at municipality level. (XLSX)
